# Recovery of a New England salt marsh six years after a natural sediment deposition event

**DOI:** 10.1371/journal.pone.0357442

**Published:** 2026-09-08

**Authors:** Gregg E. Moore, Jennifer L. Gibson, David M. Burdick, Alondra Ortiz-Franco, Ashley N. Bulseco

**Affiliations:** 1 Department of Biology, University of New Hampshire, Durham, New Hampshire, United States of America; 2 Department of Natural Resources, University of New Hampshire, Durham, New Hampshire, United States of America; 3 Natural Sciences Department, University of Puerto Rico-Cayey, Cayey, Puerto Rico, United States of America; University of New England, UNITED STATES OF AMERICA

## Abstract

In January 2018, Winter Storm Grayson deposited a spatially heterogeneous layer of sediment across the Great Marsh on the North Shore of Massachusetts in Essex Bay, Ipswich Bay, and Plum Island. A published study of short-term responses following one growing season showed percent cover of vegetation was significantly lower in plots receiving sediment than controls, while porewater chemistry (salinity, redox potential, and sulfide concentrations) shifted slightly as biogeochemical processes developed in the new sediment. Six growing seasons post-deposition, these sites were re-evaluated to determine whether storm-delivered sediment (i) increases vegetation percent cover and species richness without suppressing plant height, (ii) influences porewater conditions, and (iii) promotes belowground live root density. Results show that vegetation cover and height were not significantly impacted by sediment thickness or year, and no interaction was found between year and sediment thickness. Salinity significantly decreased from 2018 to 2024, while interactive effects appeared for both redox potential and sulfides, largely driven by changes in plots that received 0–2 cm of sediment. Interactive effects of depth and sediment thickness on live root density appear driven by biomass in the control and 4–6 cm plots. Live root density in the top 4 cm was approximately equivalent for all core types with biomass increasing with depth until 10 cm for the plots receiving greater than 2 cm of sediment. Our results suggest that natural sediment inputs can supplement elevation and stimulate belowground biomass, particularly at thicknesses between 2–6 cm, without negatively impacting vegetation cover, species richness, or porewater chemistry at the landscape scale.

## Introduction

Coastal salt marshes are among the most effective natural systems for shoreline protection, blue-carbon storage, and habitat provisioning [1–3], yet they are increasingly vulnerable to sea-level rise, storm intensification, and associated shifts in inundation regimes [4–6]. With sea level rise, the persistence of marsh platforms depends on maintaining marsh surface elevation within a narrow tolerance relative to tidal datums through vertical accretion provided by organic matter accumulation and sediment inputs [6–8]. When this building process is disrupted, excessive flooding stress and sulfide toxicity can drive transitions from high to low marsh vegetation, panne and pool expansion, and, ultimately, marsh loss [9–13]. Episodic sediment pulses associated with coastal storms can, however, deliver “elevation capital”. Years to decades’ worth of mineral inputs can be deposited in a single event, temporarily reducing flooding stress and potentially resetting trajectories of degradation toward recovery if vegetation and edaphic conditions respond favorably [14–16].

In parallel with this natural pathway, beneficial reuse of dredge materials, commonly referred to as thin-layer placement (TLP), has emerged as a restoration strategy that deliberately supplements marsh elevation by applying a relatively shallow layer of dredged or compatible sediments to the vegetated platform [17–20]. Contemporary practice defines TLP as the addition of a thin, relatively evenly distributed sediment layer designed to raise marsh elevation while maintaining vegetation and ecosystem function [21,22]. Reported benefits of appropriately calibrated TLP include platform stabilization, reduced waterlogging and anoxia, and enhanced recovery of vegetation structure where post-placement elevations occupy the functional niche of target plant communities [23–27]. Because vegetation controls sediment capture, wave attenuation, and organic matter inputs, aboveground structure and belowground production are sentinel indicators of successful elevation supplementation [7,28]. Belowground biomass mediates sediment retention, porewater chemistry, and long-term peat development and is therefore central to assessing both the trajectory and durability of recovery following sediment addition [29].

Despite growing interest, critical knowledge gaps remain for TLP efficacy in the organic-rich marshes of New England. Most empirical studies report responses within the first one to five years following sediment addition [27,30], a window that can capture initial physiological and edaphic adjustments but may miss medium-term trajectories in root production, peat development, and vegetation composition as platforms equilibrate. Thickness sensitivity also remains debated: some studies report stronger vegetation responses at placements exceeding ~5 cm [31], while others emphasize site-specific optima spanning ~2–11 cm and caution that time horizons of a decade or more may be required for full marsh function to recover [29]. More recent syntheses underscore that the “right” thickness is contingent on starting elevation, hydrologic regime, and vegetation state, rather than a single universal target [18,32]. Furthermore, belowground responses can lag aboveground recovery and may exhibit depth-specific thresholds (e.g., biomass declines beyond ~10 cm), highlighting the need for coring-based assessments in addition to surface metrics [33].

Because large-scale TLP projects remain relatively uncommon in New England and permitting constraints can limit experimental manipulation, natural storm-driven sediment deposition events provide valuable analogs for evaluating the mechanisms and consequences of elevation supplementation at landscape scales [14,15]. Although the grain size distribution, spatial heterogeneity, and delivery mode of natural deposits can differ from engineered slurry applications, both processes can add elevation capital and modify hydroperiods in ways that should, in principle, influence vegetation dynamics, porewater chemistry, and belowground biomass through similar pathways. Natural experiments thus offer a rare opportunity to test medium-term responses across realistic gradients of thickness and habitat context in situ, and to assess potential risks (e.g., smothering or shifts in community composition) that may accompany allochthonous sediment inputs.

Here we leverage a region-wide natural experiment triggered by Winter Storm Grayson (January 2018), which deposited a spatially heterogeneous layer of mineral sediment across portions of the Great Marsh on the North Shore of Massachusetts. Across three sites within this complex (Essex, Ipswich, and Newburyport), an area on the order of tens of square kilometers received on average ~2–3 cm of sediment with local patches exceeding 4–6 cm. Immediately following deposition, we established replicated monitoring transects spanning the observed thickness gradient, including unimpacted control plots, to evaluate vegetation and edaphic responses over time [15]. We subsequently revisited the original 74 plots in 2024, enabling a medium-term (six-year) assessment of recovery trajectories.

We asked whether modest, storm-delivered sediment additions can (i) increase or maintain high-marsh vegetation cover without suppressing plant height (i.e., vigor), (ii) improve porewater conditions associated with flooding stress and anoxia—salinity, redox potential, and sulfide concentrations—consistent with hydrologic relief and oxidative recovery, and (iii) sustain or enhance belowground live root density across the upper 20 cm, particularly within the ecologically active 0–10 cm zone critical for peat accretion and shear strength [7]. Based on prior TLP studies, we hypothesized that plots receiving ≥4 cm would exhibit stronger vegetation and edaphic improvements relative to thinner additions (≤2–4 cm) and controls, reflecting greater elevation capital and reduced hydroperiod stress [29,31]. We further predicted that belowground biomass would show depth-structured responses with potential declines only at depths where added material and subsequent consolidation exceed thresholds reported in the literature [33], and that any benefits would be detectable several years post-deposition, aligning with the multi-year equilibration times suggested by process-based studies and syntheses [18,32].

By treating this storm-driven sediment pulse as an analog for thin-layer elevation enhancement, our study addresses three interrelated gaps for New England marshes: (1) the medium-term (≥5 yr) sustainability of vegetation and porewater improvements following modest elevation gains; (2) the thickness dependence of responses across control, 0–2, 2–4, and 4–6 cm strata; and (3) the coupling between surface recovery and belowground biomass profiles at centimeter resolution. Because management decisions increasingly hinge on whether small to moderate additions can stabilize high-marsh states and arrest conversion to low marsh (or open water) under contemporary sea-level rise, the outcomes reported here provide directly policy-relevant evidence for calibrating TLP targets and evaluating storm-mediated elevation capital as a complementary, sustainable pathway to resilience in New England salt marshes.

## Methods

### Site description

**Fig 1:**
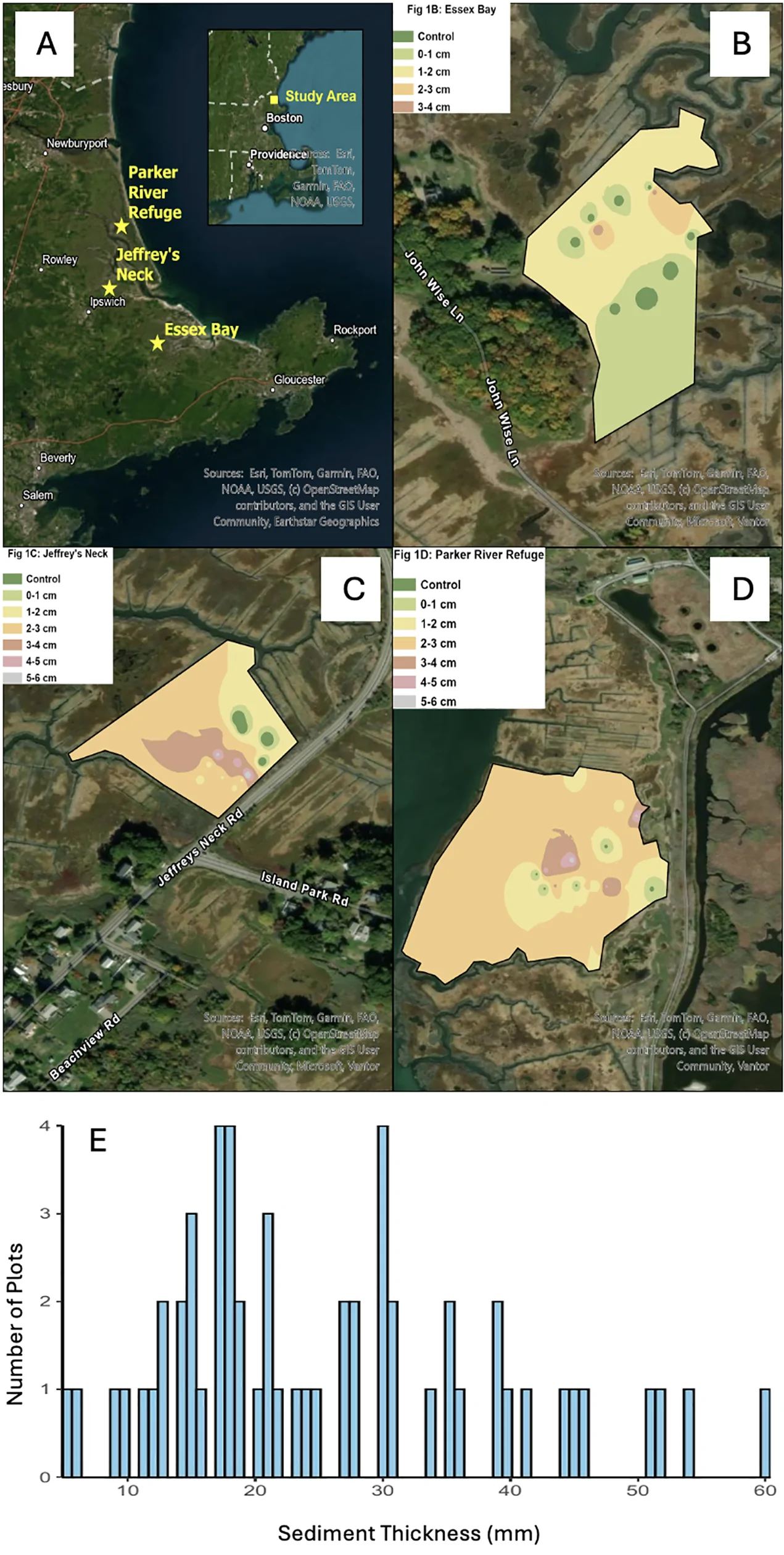
Maps of study area and histogram of sediment thickness (A) Map of approximately 40 km^2^ study site region, including (B) The Great Marsh Estuary in Essex, MA (C) Jeffrey’s Neck Road in Ipswich, MA and (D) Plum Island in Newburyport, MA, and (E) Histogram of sediment depths resulting from the natural sedimentation event in 2018.

In 2018, three sampling areas were established within the Great Marsh Estuary located on the north shore of Massachusetts that each received significant storm-delivered sediment deposition. These sampling areas occur within the same hydrologic regime and tidal elevations within Great Marsh and are dominated by the same plants (namely *Spartina alterniflora* and *S. patens*). Meanwhile, the natural sediment covered plots were found more often in habitats characterized as low marsh, transitional, and panne marsh plots. For the purposes of this analysis, they are collectively considered one site, occurring near Lowe Island in Essex, MA (42°39’16.4"N 70°46’28.3"W), Jeffery’s Neck Road in Ipswich, MA (42°41’59.5"N 70°49’06.5"W), and on Plum Island in Newburyport, MA (42°45’06.8"N 70°48’21.2"W). In total, an area approximately 40 km^2^ was covered by 2.6 ± 1.7 cm of sediment (ranging from 0 to 6 cm) due to Winter Storm Grayson (Fig 1A-E). Immediately following the event in 2018, a series of linear transects were established over the impacted areas and, along each, ten 0.5m^2^ plots were set every 20 paces (~12m) and the location recorded with a Leica GSSN Rover Real Time Kinematic (RTK) Global Positioning System (GPS) model GS14 [15]. Access to these study sites was granted by the property owners, including The Trustees of Reservations for Lowe Island and Jeffrey’s Neck, and the US Fish and Wildlife Service Parker River National Wildlife Refuge for the Plum Island site. No permits were required for the work completed. In the summer of 2019 (July) and 2024 (July and September), 74 of the previously studied sediment deposition plots were revisited for vegetation and porewater monitoring. Of the plots revisited, 17 were designated as controls (no sediment addition) and the rest were categorized as 0–2 cm (n = 25), 2–4 cm (n = 24) or 4–6 cm (n = 8) sediment addition that occurred due to natural deposition. Thickness was determined in the winter of 2018 immediately following the Winter Storm Grayson using a meter stick inserted into the deposited sediment [15]. We recognize sediment addition depths may partially reflect underlying elevation gradients rather than sediment addition alone. However, the mean elevation of control plots was equal to or less than that of experimental plots. It may be important to note that control plots had a greater percentage of *S. patens* and *Distichlis spicata*, which was to be expected as they most were characterized as true high marsh plots.

### Field collection and laboratory analyses

#### Vegetation assessment.

Vegetation sampling was completed during the growing season to represent peak plant growth for each of the three years of the study. Percent cover was recorded using visual estimation within a 0.5m^2^ PVC quadrat assuming a single canopy layer (maximum of 100%) at each plot (total n = 74). Observers were trained using calibrated images to visually estimate percent cover. Vascular plants were identified to species level in the field and mean canopy height (cm) was measured in 2019 and 2024 (mean of 3 haphazard measures, taken from the base of the plant to the terminus of an individual’s longest leaf). Bare/unvegetated ground was accounted for in the percent cover estimate. Analysis of percent cover was focused on comparison of total coverage changes over time and not species-specific changes *per se*. Species richness was calculated as the number of vascular plant species per plot.

#### Porewater analyses.

Soil porewater was taken concurrently with vegetation studies in 2018, 2019, and 2024, and sampled at a depth of ~25 cm at each plot within approximately 2 hours (+/-) of low tide using the sipper method [34,35], which extracts water held in pore spaces using a 1 mm diameter stainless steel tube fitted with a 60cc plastic syringe. The depth of ~25 cm is consistently within the original marsh surface, accessing porewater associated with live roots of the existing plant community. Approximately 10mL of porewater was expelled into a 25mL polytetrafluoroethylene (PTFE) vial for field determination of salinity and redox, while 500 μL of porewater was subsampled into an additional vial containing 12mL of 2% Zinc Acetate solution as a fixative for sulfide analysis later in the lab. Porewater salinity was determined in the field using a Thermo Scientific Orion Star A329 Portable Multiparameter Meter with DuraProbe conductivity cell, while redox potential was obtained using a platinum electrode (Thermo Fisher Scientific, Waltham, MA). Zinc acetate fixed samples were stored at 4^o^C in the laboratory for analysis of sulfide content (mM) using Cline’s [36] colorimetric analysis and a LaMotte SmartSpec (Chestertown, MD, USA) spectrophotometer.

#### Root biomass assessment.

In 2024, peat cores were exhumed from each of the 74 plots to a depth of 20 cm using a 1m Eijkelkamp (TM) gouge auger with 30 mm diameter bore for analysis of root biomass. Cores were wrapped in aluminum foil and placed in Ziploc® bags in the field and promptly stored in the laboratory at 4^o^C until processing. To determine root biomass, cores were sectioned into ten segments by depth every 2 cm. Although 20 cm cores were exhumed, the terminus of some cores was incomplete when examined in the lab (*i.e.,* full volume of the base of the core was partially missing). As a result, the bottom ~2 cm was removed to ensure full core volume for all analyses, resulting in a final core length of 18 cm. Each segment was placed in a stainless steel 1.00 mm sieve and rinsed with water to separate the roots and rhizomes from other organic and inorganic peat core constituents. The resulting organic material was sorted as live or dead and placed into pre-weighed tin weighing boats and dried at 45^o^C for 2–3 days until mass was consistent between measurements and dry weight (g) was recorded. Live root density (mg/cm^3^) was calculated from the dry weight of the live roots/rhizomes divided by the volume of the 2 cm core section (r = 1.5 cm). Areal root biomass (g/m^2^) was determined by summing all belowground biomass (live/dead) and dividing by area (r = 0.015 m).

### Statistical analyses

Data were managed in MS Excel and analyzed and graphed using R 4.5.2 scripts [37]. When original and parametrically transformed data did not meet the assumptions of normality and homoscedasticity, aligned rank transformations (ART) were used, otherwise parametric tests were performed. Unlike standard rank transformation that simply ranks the response variable and does not properly take interactions into account, ART first aligns the data with respect to the effect being tested prior to ranking. Where interactive effects may be present, type III tests (mixed effects) were run to account for the interactive effects. To explore significant differences when present, estimated marginal means were used and the Tukey method was applied to adjust p-values for family-wise error across four or three group comparisons. For all tests, a significance level of α = 0.05 was used.

Prior to analysis, percent cover of all vascular plants within a plot was summed and converted from percentages to proportions. To compensate for plots with 0 or 100% total vascular plant coverage, proportions were transformed using (*y*(*n*-1)+0.5)/*n*, where *y* was the proportion and *n* was the total number of plots visited across all years and sediment thicknesses [38]. The proportions were then analyzed using a beta regression model with a logit link function [39] where year (2018, 2019, and 2024), sediment thickness (Control, 0–2 cm, 2–4 cm, and 4–6 cm), and the interaction of the two were fixed effects, with the reference being control treatments in 2018. To obtain total vascular percent cover by year or sediment thickness, data were back-transformed using estimated marginal means. An ART for factorial models [40] with a type III Anova (mixed effects), where plot was the random variable, was used to investigate the main and interactive effects of the fixed variables year (vegetation height: 2019 and 2024; porewater chemistry: 2018, 2019, and 2024) and sediment thicknesses (Control, 0–2, 2–4, 4–6) on vegetation height, as well as the porewater chemistry parameters: salinity, sulfide concentration, and redox.

To assess the main and interactive effects of year and sediment thickness on species richness, a generalized linear mixed model with a Conway-Maxwell-Poisson distribution was used to account for underdispersion in the count data [41]. A type III Wald chi-square test was run to test for statistical significance where year (2018, 2019, and 2024) and sediment thickness were fixed effects and plot was the random effect to account for repeated measurements over the years. To ensure assumptions were met, simulation-based residual plots and dispersion were tested using the DHARMa package [42].

To investigate the main and interactive effects of sediment thickness at different depths on live root biomass, a general linearized mixed model with Tweedie distribution and a log link function was used, due to the data having zeros and being right-skewed, where “core” was set as the random effect [43]. The effects of sediment thickness on areal root biomass were then analyzed using a one-way ANOVA. Cores were only taken in 2024, so year was not a factor.

## Results

### Vegetation

**Fig 2:**
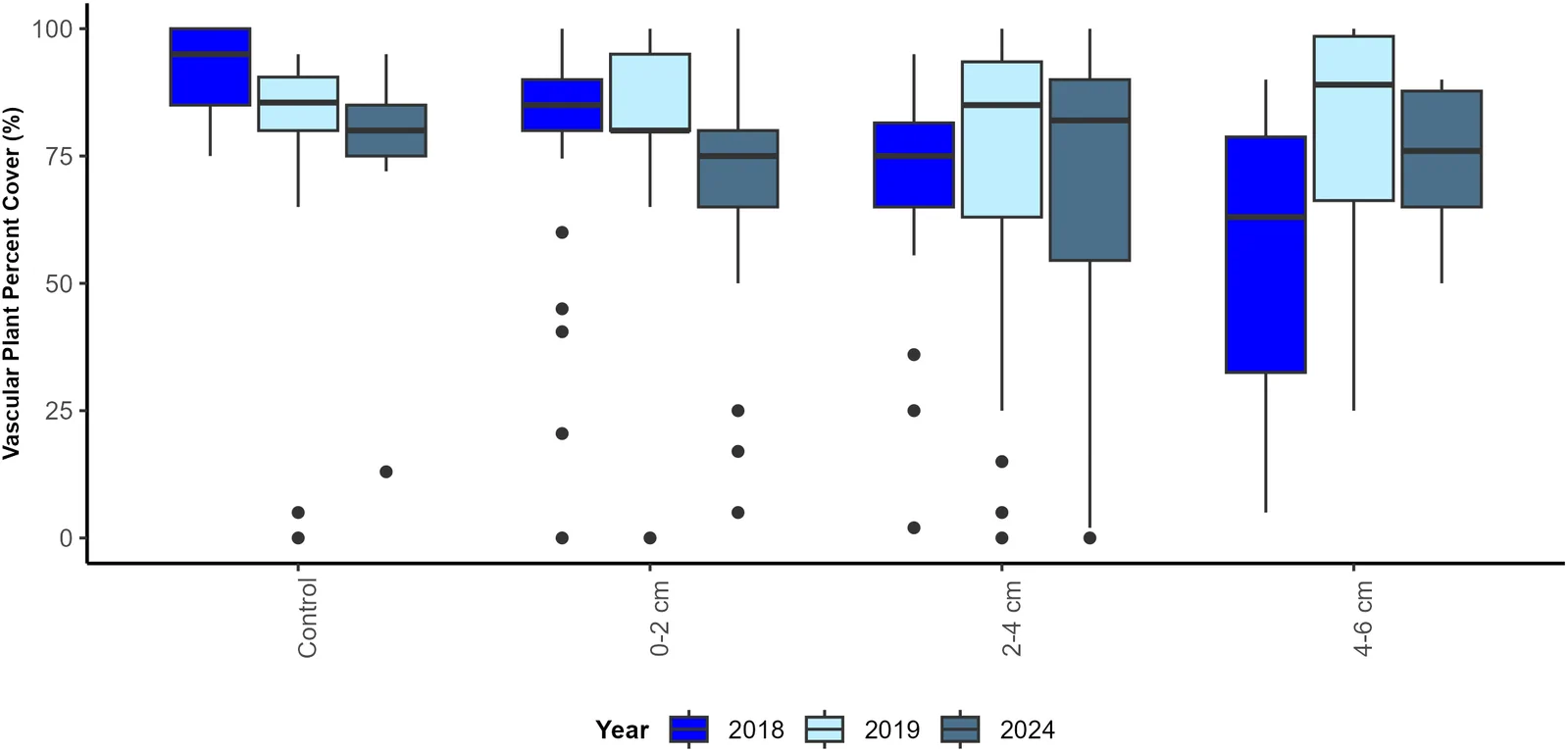
Total vascular plant percent cover by sediment thickness for years studied. Details on individual species cover can be found in S1 Table.

Our plots included a total of six plant species that were found in each year studied (2018, 2019, & 2014): *Spartina alterniflora* (predominantly short-form)*, S. patens*, *Distichlis spicata*, *Salicornia depressa*, *Limonium nashii*, and *Suaeda maritima*, with *Atriplex patula* appearing in 2024 (S1 Table). From 2018 to 2019, control treatments experienced a significant decline in total vascular plant percent cover from 83.9% to 67.2% (z = 2.513, p = 0.032) with little change (+1.6%) from 2019 to 2024 (68.8%; z = −0.202, p = 0.978). The overall decline from 2018 to 2024 in control treatments was only marginally significant (z = 2.312, p = 0.054). With the addition of sediment from the storm in 2018, vascular plant percent cover was significantly lower in both 2–4 cm (63.6%; z = 3.177, p = 0.008) and 4–6 cm (53.7%; z = 3.227, p = 0.007) sediment thickness plots, but not significantly lower in 0–2 cm treatments (70.2%; z = 2.338, p = 0.090) when compared to control treatments. In 2019 and 2024, no significant differences were noted between any sediment thickness treatments (2019: p > 0.473; 2024: p > 0.952). Although covered with sediment in 2018, all sediment addition plots rebounded within a year and appeared to retain their plant cover through 2024 (Fig 2).

**Fig 3:**
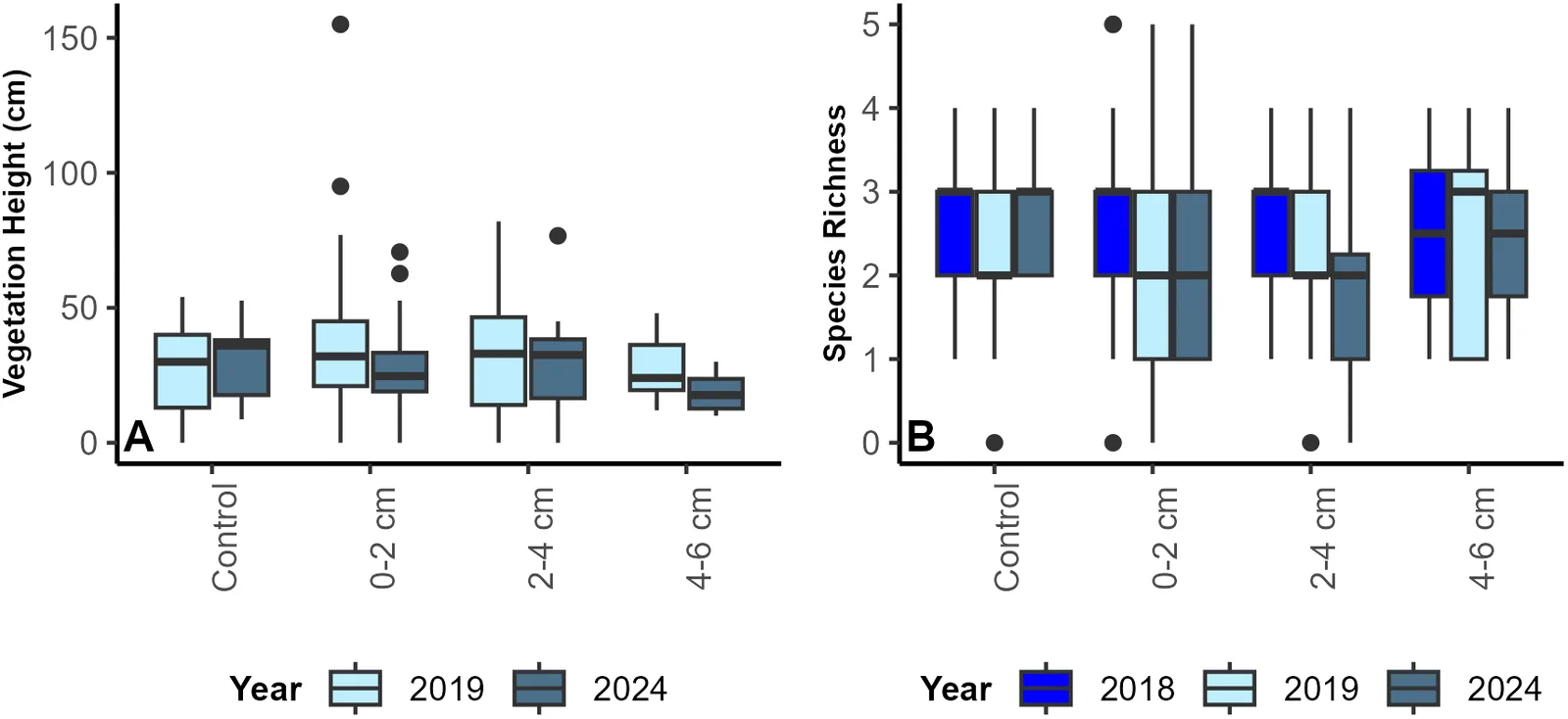
(A) Vegetation height (cm) and (B) Species richness per plot by sediment thickness (control, 0-2 cm, 2-4 cm, 4-6 cm) for years studied.

Vegetation height, only measured in 2019 and 2024, did not appear to be significantly impacted by sediment thickness (F_3, 70_ = 0.739, p = 0.532, Fig 3A) or year (F_1, 70_ = 1.538, p = 0.219), with no interaction found between year and sediment thickness (F_3, 70_ = 0.930, p = 0.431). Although an additional species was found in 2024 (*A. patula*), no significant change in species richness (Fig 3B) was noted for year (x^2^_2_ = 2.332, p = 0.312), different sediment thicknesses (x^2^_2_ = 0.617, p = 0.892), or the interactive effect of year and thickness (x^2^_2_ = 11.438, p = 0.076). Although not significant (p > 0.05), mean species richness dropped marginally from 2.66 ± 0.12 in 2018, to 2.23 ± 0.13 in 2019, and remained relatively unchanged from 2019 to 2024 (2.20 ± 0.12)*.*

### Porewater

**Fig 4:**
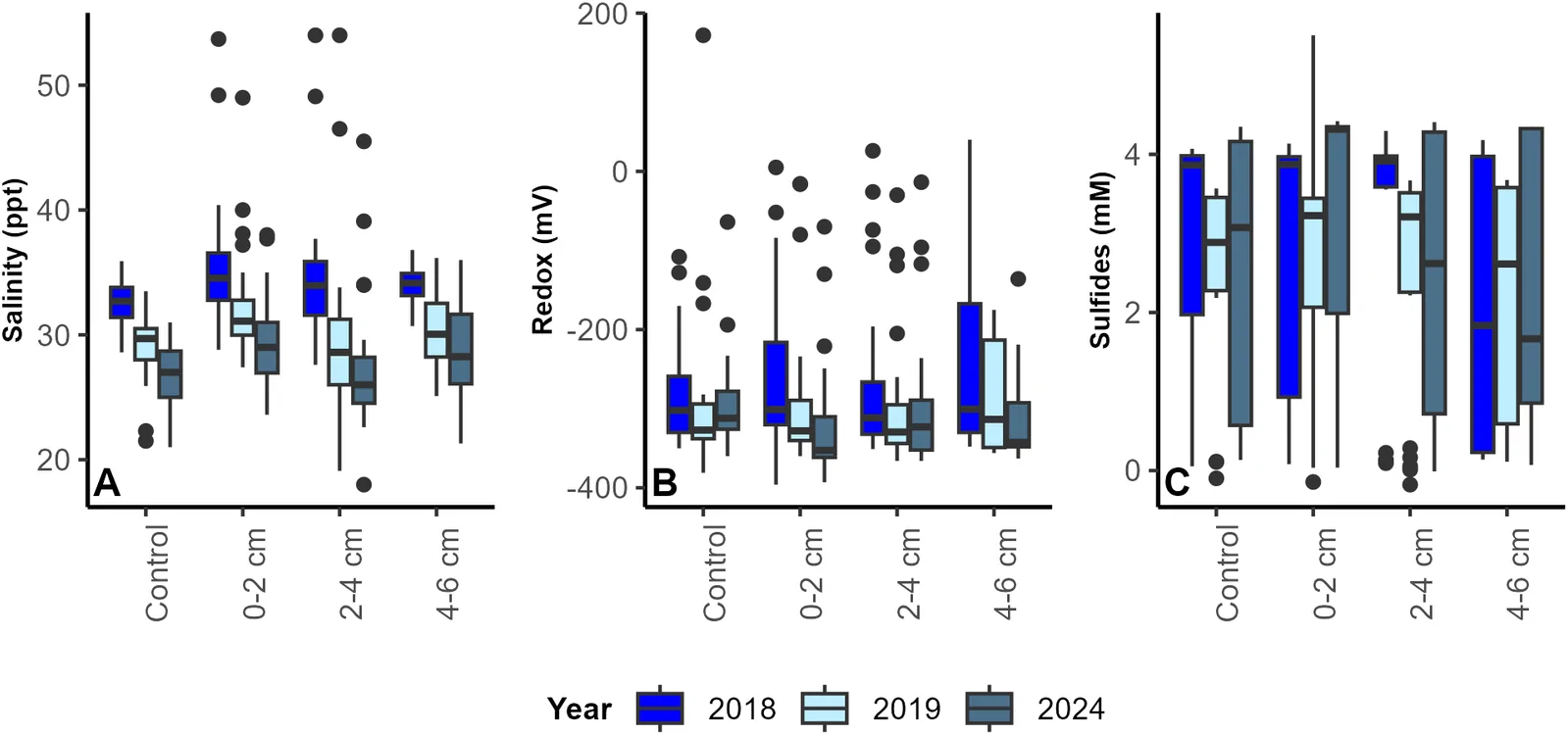
Salinity (A), redox potential (B), and sulfide concentrations (C) for sediment thicknesses over the years studied.

No interactive effects appeared in the analysis of salinity levels (F_6,138_ = 0.685, p = 0.662), but year pooled over sediment thickness (F_2,138_ = 107.660, p < 0.001; Table 1; Fig 4A) and sediment thickness pooled over years (F_3,69_ = 3.551, p = 0.019) had significant effects. Salinity significantly decreased from 2018 to 2019 (t_138_ = 9.195, p < 0.001) and again from 2019 to 2024 (t_138_ = 5.306, p < 0.001). Additionally, plots that received 0–2 cm sediment had significantly higher salinity than control plots (t_69_ = −2.828, p = 0.031) and marginally higher salinity than 2–4 cm plots (t_69_ = 2.639, p = 0.049). Interactive effects of year and sediment thickness appeared for both redox potential (F_6,138_ = 2.550, p = 0.023) and sulfide concentration (F_6,136_ = 3.076, p = 0.007). The interactive effect for redox was driven by the drop in redox at 0–2 cm sediment thickness plots from 2018 to 2024 (t_115_ = 6.178, p < 0.001; Fig 4B; Table 1) whereas redox remained relatively stable throughout the years for other sediment thicknesses (p > 0.113). The interactive effects for sulfides were again primarily driven by changes to sulfides in the 0–2 cm sediment thickness plots where sulfides increased significantly from 2018 to 2024 (t_136_ = −3.485, p = 0.031) and from 2019 to 2024 (t_136_ = −6.022, p < 0.001; Fig 4C; Table 1). Additionally, in 2024, sulfides in-0–2 cm sediment thickness plots were significantly higher than sulfides in both 2–4 cm sediment thickness (t_106_ = −3.748, p = 0.015) and control plots (t _106_ = −3.578, p = 0.025) in 2019. The only other significant difference noted for sulfides was in 2–4 cm sediment thickness plots between 2018 and 2019 (t _136_ = 4.103, p = 0.004).

**Table 1:** Porewater metrics for each plot type over years with mean ± standard error.

		2018	2019	2024
Salinity (ppt)	Control	32.43 ± 0.51	28.77 ± 0.75	26.52 ± 0.65
	0-2 cm	35.78 ± 1.17	32.37 ± 0.97	29.38 ± 0.79
	2-4 cm	34.53 ± 1.23	29.89 ± 1.48	27.48 ± 1.18
	4-6 cm	34.01 ± 0.73	30.35 ± 1.40	28.84 ± 1.81
Redox (mV)	Control	−275.18 ± 17.89	−279.18 ± 32.04	−289.47 ± 17.48
	0-2 cm	−254.25 ± 21.25	−285.46 ± 20.67	−317.74 ± 16.11
	2-4 cm	−264.71 ± 22.39	−293.04 ± 18.14	−292.07 ± 18.83
	4-6 cm	−229.75 ± 51.87	−282.88 ± 27.58	−302.38 ± 28.72
Sulfides (mM)	Control	2.79 ± 0.39	2.49 ± 0.31	2.47 ± 0.44
	0-2 cm	2.84 ± 0.34	2.60 ± 0.30	3.23 ± 0.35
	2-4 cm	3.10 ± 0.33	2.48 ± 0.29	2.55 ± 0.37
	4-6 cm	2.05 ± 0.31	2.14 ± 0.55	2.20 ± 0.67

### Cores

**Fig 5:**
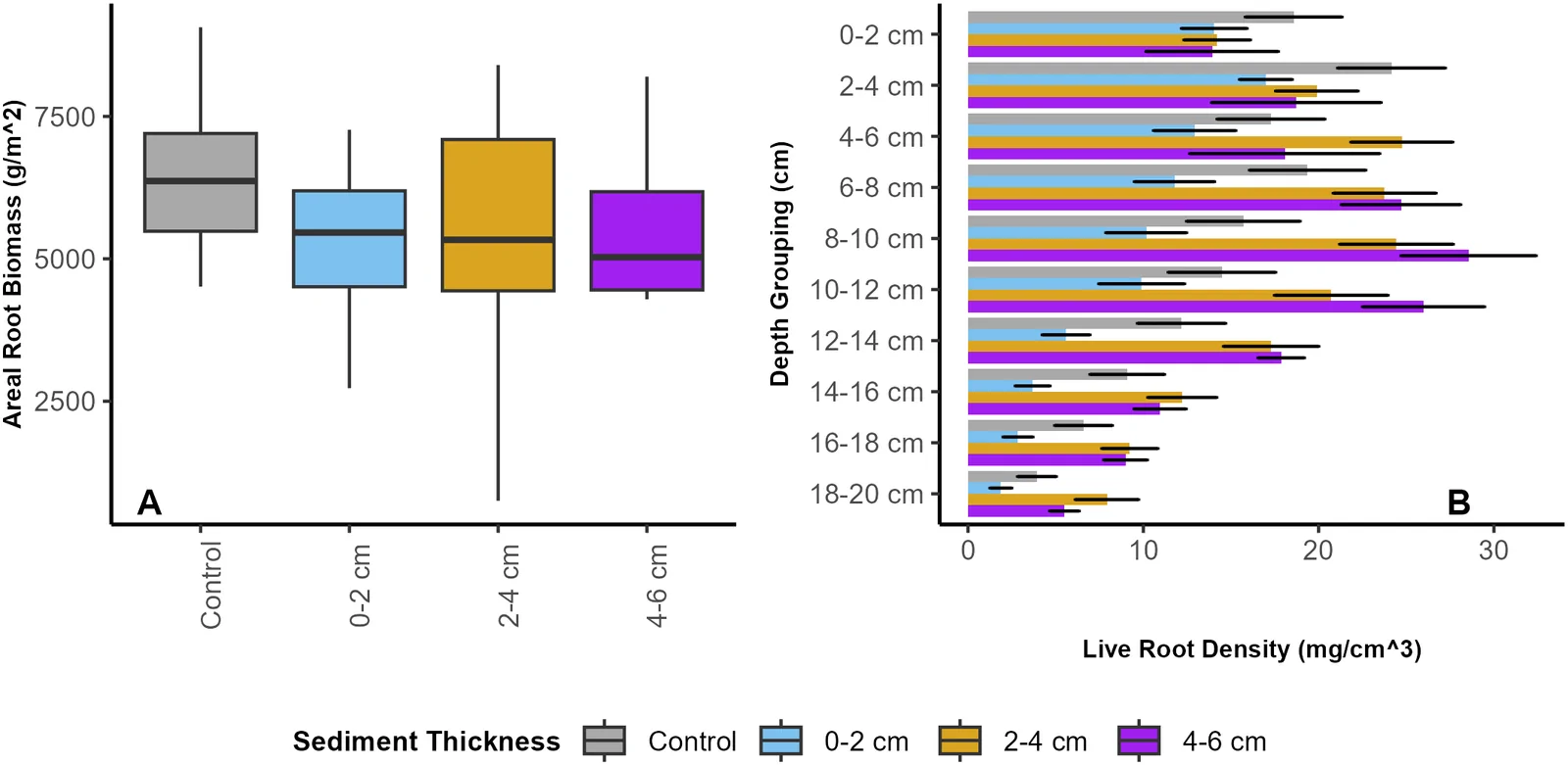
(A) Areal biomass (B) Live root density over depth for each sediment thickness type.

Areal root biomass in 2024 did not significantly differ between sediment thicknesses (F_3, 70_ = 1.768, p = 0.161; Fig 5A). However, live root density over depth for the different sediment thicknesses was subject to an interactive effect (x^2^_24_ = 86.21, p < 0.001; S2 Table; Fig 5B). When estimated marginal means were examined to compare sediment thicknesses by depth, we found no difference between sediment thickness within the first 4 cm, but after 4 cm, the live root biomass in the 0–2 cm sediment thickness plots dropped significantly by over 40% from 2–4 cm sediment thickness plots (z = −2.818, p = 0.025) and, at the 6–8 cm depth, the 0–2 cm sediment thickness plots remained significantly lower than the 2–4 cm (z = −2.946, p = 0.017) and 4–6 cm (z = −2.710, p = 0.034) sediment thickness plots (S2 Table; Fig 5B). For all other sediment depths, the 0–2 cm sediment thickness continued to have significantly lower live root biomass than the 2–4 cm treatment (at 8–10 cm: z = −3.749, p = 0.001; at 10–12 cm: z = −3.091, p = 0.012; at 12–14 cm: z = −4.322, p = 0.001, at 14–16 cm: z = −4.329, p < 0.001; at 16–18 cm: z = −4.128, p < 0.001) and the 4–6 cm treatment (at 8–10 cm depth: z = −3.734, p = 0.001; at 10–12 cm depth: z = −3.513; p = 0.003; at 12–14 cm: z = −3.907, p = 0.001; at 14–16 cm: z = −3.485, p = 0.003; at 16–18 cm: z = −3.550, p = 0.002). Over all depths, no significant difference was seen between the two greater sediment thicknesses (2–4 cm and 4–6 cm) or between those two thicknesses and controls. However, after 12 cm and for all deeper depths, control plots exhibited significantly more biomass than the 0–2 cm sediment thickness plots (at 12–14 cm: z = 2.795, p = 0.027; at 14–16 cm: z = 2.976, p = 0.016, at 16–18 cm: z = 2.655, p = 0.040).

## Discussion

### Vegetation responses indicate no long-term negative effects

Sediment deposition events may have short-term, negative effects on vegetation by burying shoots, reducing light availability, altering porewater chemistry, and/or limiting gas exchange [31,44,45], however our study found no long-term negative effects on plant communities. While plant cover [15] and species richness (this study) decreased in the first growing season after the storm (2018–2019; Fig 2), both metrics were not significantly different from control plots thereafter (from 2019 and 2024). In addition, there were no differences in vegetation height and belowground biomass over medium time scales, between 2019–2024 (Fig 3A-B). This immediate, short-term decline has been observed in other studies as well. For example, in a study where ~23 mm of dredged material was sprayed onto a marsh in coastal Louisiana, measurements made immediately (~3 weeks) following treatment revealed diminished stem counts in *S. alterniflora,* but then largely recovered by the following year [46]. Payne et al. [24] observed fewer stems in *S. patens* two months after experimental units were amended with 10 cm sand when compared to controls, suggesting this high marsh species is less adapted to porewater conditions that may result from sediment deposition [11,47]. Sediment additions of 7 and 14 cm in Raposa et al. [26] resulted in early reductions in percent cover in both low and high marsh habitat but by the end of three years found that there was no difference in TLP versus control plots, demonstrating plant growth following sedimentation. Likewise, Puchkoff & Lawrence [25] observed 89% fewer stems in sediment treated plots (+5, + 10, and +15 cm) controls but found that sediment thicknesses of 5–7 cm resulted in similar vegetation to controls after one growing season. Despite these initial early reductions, plant communities seemed to transition into a period of sustained regrowth, possibly driven by root production [22] or enhanced nutrient delivery [31,43], following the immediate stress of sediment burial. This is supported by our results, where both vegetation height and species richness were the same between controls, 0–2 cm, 2–4 cm, and 4–6 cm between 2019 and 2024, following the initial negative response in year one.

In addition to aboveground plant communities, there was no significant difference in belowground biomass when comparing the different sediment thicknesses to controls (Fig 5A). This is likely because sediment addition helps to build the marsh platform, which may improve soil aeration and encourage root proliferation. Collectively, these changes may improve porewater conditions that support plant productivity and uptake of carbon dioxide [48,49]. Our results are consistent with previous work suggesting that sediment addition allows for the formation of new root and rhizomes as a result of rapid expansion into empty space [22,50], demonstrating the importance of belowground biomass growth as a mechanism for plant recovery in response to burial [51]. Payne et al. [24], for instance, found that total biomass and root mass were not impacted by 10 cm of added sand to either *S. alterniflora* or *S. patens*, and Puchkoff & Lawrence [25] observed a 149% increase in root density in response to 5–7 cm of sediment addition. Similarly, Cheng et al. [30] found that live root density was ~ 3x higher in soils where a sediment slurry was added relative to the control six years later, attributing this pattern to nutrient limitation. Given root growth seems to be an important mechanism of plant recovery in response to sediment deposition, we argue that monitoring belowground biomass and overall root growth will be an important consideration in assessing coastal wetland resilience.

In other regions of the U.S., there has been strong evidence for positive responses by vegetation to sediment addition. In a mesocosm experiment in Virginia, USA, *S. alterniflora* total biomass increased by up to 120% with an optimal burial depth of 5–10 cm [51], slightly greater than thicknesses observed in our study. *S. alterniflora* primary productivity increased by 20–60% in response to hurricane-induced sedimentation of ~5–10 cm in Louisiana [48] and aboveground plant biomass increased by ~50% following a 3–8 cm deposition in two marshes in the Mississippi River deltaic basins as a result of Hurricane Katrina [52]. In the Gulf of Mexico, a sediment slurry addition led to increased soil fertility and plant growth [43], and in Louisiana, areas that received up to 60 cm (47–94 kg m^2^) of sediment exhibited significant increases in leaf area, aboveground biomass, number of new culms, and transpiration rates approximately two years following when compared to non-sediment controls [53]. Although we did not detect clear positive responses in the plant community after six years, the fact that all plots exhibited comparable plant height, richness, and belowground biomass suggests that sediment addition did not hinder vegetation recovery in this New England salt marsh and that longer time scales (>15 years) could be required to understand any beneficial effects that emerge, as is common in restored wetlands [18,29,54,55].

### Porewater chemistry

Sediment deposition beyond optimal depths [51] has the potential to disrupt porewater chemistry by limiting oxygen diffusion into the rooting zone, thus enhancing anerobic metabolic activity and promoting short-term accumulation of reduced compounds such as sulfides and ammonium that may inhibit plant growth and uptake of essential nutrients [44,45,56,57]. However, our results did not show evidence of these negative shifts, suggesting that the sediment additions resulting from storm deposition did not exceed the system’s capacity to maintain stable porewater chemistry. Throughout our six-year study, porewater salinity decreased significantly from 2018 to 2024, with 0–2 cm exhibiting the highest salinities when compared to 2–4 cm and the control plots (Fig 4A). Small increases in elevation (cm scale) resulting from sediment deposition may have reduced flooding and improved drainage, as observed in other studies [31,43], resulting in lower salinity. However, because salinity declined consistently across all treatments over time, this downward trend is more likely a result of landscape-scale hydrologic conditions and high precipitation during our time of sampling in 2024 [58]. Salinity was highest in the 0–2 cm range potentially because the sediment inputs were insufficient to alter elevation or drainage and may have even facilitated increased evaporation or salt accumulation near the surface of the marsh platform. This pattern coincided with elevated sulfide and lower redox potential relative to the other sediment thicknesses, suggesting that very thin sediment deposits may provide limited benefits to porewater chemistry. Prior studies have similarly found that responses to sediment addition can be thickness-dependent, with thinner deposits producing smaller improvements in elevation, drainage, and redox conditions than moderate sediment additions [17]. However, increases in sulfide concentrations over time may also reflect the recovery of a more natural salt marsh community. Greater plant productivity and organic matter inputs stimulate sulfate reduction, which produces sulfide along with other microbial metabolic byproducts [59]. The decline in sulfide concentrations in the 2–4 cm plots from 2018–2019 may reflect these processes. For example, sediment deposition or freshwater inputs can increase iron availability, promoting the precipitation of dissolved sulfide, thereby lowering sulfide concentrations following sediment burial [60,61]. Overall, these results suggest that sediment deposition did not adversely affect porewater chemistry and rather, may have helped to re-establish typical geochemical conditions of a natural New England salt marsh.

## Conclusion

We leveraged a region-wide, natural experiment triggered by Winter Storm Grayson, which deposited a layer of spatially heterogeneous sediment across portions of the Great Marsh estuary on the North Shore of Massachusetts, USA. We evaluated vegetation and edaphic responses to sediment delivery over a medium-term (> 5 year) time scale [15] and treated this storm-driven sediment deposition as an analog for thin-layer elevation supplementation to show sediment additions can 1) increase or maintain high-marsh vegetation cover without suppressing plant height, 2) improve porewater conditions associated with flooding stress and anoxia, and 3) sustain or enhance belowground live root density across the upper 20 cm and particularly the active 0–10 cm zone, which is most critical for peat accretion and shear strength. We also showed that live root density was greatest in the 2–4 and 4–6 sediment thickness ranges, which suggests there may be an optimal range for maximizing plant recovery and enhancing marsh resilience [51]. Overall, our results suggest that sediment inputs, even when spatially patchy, can supplement elevation without negatively impacting vegetation and may instead promote belowground biomass stabilization and marsh recovery at the landscape scale.

## Supporting information

S1 Table Percent cover (mean ± standard error) by species in different plot type over the years. (DOCX)

S2 Table Pairwise comparisons for live root biomass (mg/cm^3^) over depth. (DOCX)
