# Supplementary material for: Recovery of a New England salt marsh six years after a natural sediment deposition event
Source: PLoS One. 2026 Sep 8;21(9):e0357442. doi: 10.1371/journal.pone.0357442 (PMC13552792; doi:10.1371/journal.pone.0357442)
Supplement: Supplementary file 1 [file pone.0357442.s001.docx]

| **S1 Appendix: Percent cover (mean ± standard error) by species in different plot type over the years.** | | | | |
| --- | --- | --- | --- | --- |
|  | | **2018** | **2019** | **2024** |
| *S. alternifora* | Control | 16.2 ± 4.2 | 16.9 ± 5.8 | 14.5 ± 3.8 |
|  | 0-2 cm | 42.2 ± 7.2 | 50.0 ± 7.1 | 42.0 ± 5.9 |
|  | 2-4 cm | 23.4 ± 4.8 | 23.3 ± 5.9 | 24.3 ± 6.3 |
|  | 4-6 cm | 20.6 ± 5.7 | 33.3 ± 9.7 | 28.4 ± 8.6 |
| *S. patens* | Control | 52.4 ± 6.4 | 38.3 ± 6.4 | 47.9 ± 4.1 |
|  | 0-2 cm | 20.0 ± 4.6 | 23.5 ± 5.7 | 22.1 ± 5.4 |
|  | 2-4 cm | 32.8 ± 5.8 | 38.7 ± 7.9 | 36.5 ± 7.6 |
|  | 4-6 cm | 16.3 ± 8.3 | 21.2 ± 10.8 | 28.8 ± 10.1 |
| *D. spicata* | Control | 20.8 ± 6.2 | 20.7 ± 5.7 | 14.4 ± 3.5 |
|  | 0-2 cm | 12.2 ± 3.9 | 7.4 ± 2.7 | 3.9 ± 1.6 |
|  | 2-4 cm | 12.1 ± 3.4 | 9.1 ± 2.8 | 7.3 ± 2.1 |
|  | 4-6 cm | 16.3 ± 6.7 | 22.4 ± 10.2 | 16.9 ± 8.3 |
| *S. depressa* | Control | 0.4 ± 0.3 | 0.1 ± 0.1 | 0.1 ± 0.1 |
|  | 0-2 cm | 1.7 ± 0.7 | 0.4 ± 0.2 | 0.3 ± 0.2 |
|  | 2-4 cm | 1.2 ± 0.4 | 1.2 ± 0.7 | 0.5 ± 0.3 |
|  | 4-6 cm | 2.6 ± 1.4 | 0.1 ± 0.1 | 0.9 ± 0.9 |
| *S. maritima* | Control | 0.1 ± 0.1 | 0.03 ± 0.0 | 0.1 ± 0.1 |
|  | 0-2 cm | 0.6 ± 0.6 | 0 ± 0 | 0.1 ± 0.1 |
|  | 2-4 cm | 0.2 ± 0.2 | 0 ± 0 | 0 ± 0 |
|  | 4-6 cm | 0 ± 0 | 0 ± 0 | 0 ± 0 |
| *L. nashii* | Control | 1.5 ± 1.5 | 0 ± 0 | 0.1 ± 0.1 |
|  | 0-2 cm | 0.04 ± 0.03 | 0.1 ± 0.1 | 0.1 ± 0.1 |
|  | 2-4 cm | 0.02 ± 0.02 | 0 ± 0 | 0 ± 0 |
|  | 4-6 cm | 0 ± 0 | 0 ± 0 | 0 ± 0 |
| *A. patula* | Control | 0 ± 0 | 0 ± 0 | 0 ± 0 |
|  | 0-2 cm | 0 ± 0 | 0 ± 0 | 0.4 ± 0.4 |
|  | 2-4 cm | 0 ± 0 | 0 ± 0 | 0 ± 0 |
|  | 4-6 cm | 0 ± 0 | 0 ± 0 | 0 ± 0 |
