# Supplementary material for: Recovery of a New England salt marsh six years after a natural sediment deposition event
Source: PLoS One. 2026 Sep 8;21(9):e0357442. doi: 10.1371/journal.pone.0357442 (PMC13552792; doi:10.1371/journal.pone.0357442)
Supplement: Supplementary file 2 [file pone.0357442.s002.docx]

| **S2 Appendix: Pairwise comparisons for live root biomass (mg/cm^3^) over depth** | | | | | |
| --- | --- | --- | --- | --- | --- |
| **Depth** | **Treatment** | **Treatment** | **Ratio** | **Z** | **p-value** |
| 0-2 cm | Control | 0-2 cm | 1.279 | 0.891 | 0.810 |
|  | Control | 2-4 cm | 1.296 | 0.929 | 0.790 |
|  | Control | 4-6 cm | 1.196 | 0.469 | 0.966 |
|  | 0-2 cm | 2-4 cm | 1.013 | 0.050 | 1.000 |
|  | 0-2 cm | 4-6 cm | 0.934 | -0.186 | 0.998 |
|  | 2-4 cm | 4-6 cm | 0.923 | -0.220 | 0.996 |
| 2-4 cm | Control | 0-2 cm | 1.355 | 1.131 | 0.670 |
|  | Control | 2-4 cm | 1.234 | 0.780 | 0.863 |
|  | Control | 4-6 cm | 1.206 | 0.509 | 0.957 |
|  | 0-2 cm | 2-4 cm | 0.911 | -0.376 | 0.982 |
|  | 0-2 cm | 4-6 cm | 0.891 | -0.328 | 0.988 |
|  | 2-4 cm | 4-6 cm | 0.977 | -0.065 | 1.000 |
| 4-6 cm | Control | 0-2 cm | 1.382 | 1.150 | 0.658 |
|  | Control | 2-4 cm | 0.680 | -1.404 | 0.497 |
|  | Control | 4-6 cm | 0.849 | -0.437 | 0.972 |
|  | **0-2 cm** | **2-4 cm** | ***0.492*** | ***-2.818*** | ***0.025*** |
|  | 0-2 cm | 4-6 cm | 0.614 | -1.359 | 0.526 |
|  | 2-4 cm | 4-6 cm | 1.248 | 0.627 | 0.923 |
| 6-8 cm | Control | 0-2 cm | 1.691 | 1.867 | 0.242 |
|  | Control | 2-4 cm | 0.799 | -0.819 | 0.845 |
|  | Control | 4-6 cm | 0.654 | -1.163 | 0.650 |
|  | **0-2 cm** | **2-4 cm** | ***0.473*** | ***-2.946*** | ***0.017*** |
|  | **0-2 cm** | **4-6 cm** | ***0.387*** | ***-2.710*** | ***0.034*** |
|  | 2-4 cm | 4-6 cm | 0.819 | -0.581 | 0.938 |
| 8-10 cm | Control | 0-2 cm | 1.656 | 1.745 | 0.300 |
|  | Control | 2-4 cm | 0.628 | -1.671 | 0.339 |
|  | Control | 4-6 cm | 0.448 | -2.199 | 0.123 |
|  | **0-2 cm** | **2-4 cm** | ***0.379*** | ***-3.749*** | ***0.001*** |
|  | **0-2 cm** | **4-6 cm** | ***0.270*** | ***-3.734*** | ***0.001*** |
|  | 2-4 cm | 4-6 cm | 0.714 | -0.991 | 0.755 |
| 10-12 cm | Control | 0-2 cm | 1.555 | 1.512 | 0.430 |
|  | Control | 2-4 cm | 0.693 | -1.303 | 0.561 |
|  | Control | 4-6 cm | 0.450 | -2.168 | 0.132 |
|  | **0-2 cm** | **2-4 cm** | ***0.445*** | ***-3.091*** | ***0.012*** |
|  | **0-2 cm** | **4-6 cm** | ***0.290*** | ***-3.513*** | ***0.003*** |
|  | 2-4 cm | 4-6 cm | 0.650 | -1.250 | 0.595 |
| 12-14 cm | **Control** | **0-2 cm** | ***2.366*** | ***2.795*** | ***0.027*** |
|  | Control | 2-4 cm | 0.711 | -1.185 | 0.637 |
|  | Control | 4-6 cm | 0.553 | -1.563 | 0.400 |
|  | **0-2 cm** | **2-4 cm** | ***0.300*** | ***-4.322*** | ***0.001*** |
|  | **0-2 cm** | **4-6 cm** | ***0.234*** | ***-3.907*** | ***0.001*** |
|  | 2-4 cm | 4-6 cm | 0.777 | -0.708 | 0.894 |
| 14-16 cm | **Control** | **0-2 cm** | ***2.655*** | ***2.976*** | ***0.016*** |
|  | Control | 2-4 cm | 0.733 | -1.030 | 0.732 |
|  | Control | 4-6 cm | 0.662 | -1.028 | 0.733 |
|  | **0-2 cm** | **2-4 cm** | ***0.276*** | ***-4.329*** | ***<0.001*** |
|  | **0-2 cm** | **4-6 cm** | ***0.249*** | ***-3.485*** | ***0.003*** |
|  | 2-4 cm | 4-6 cm | 0.903 | -0.272 | 0.993 |
| 16-18 cm | Control | 0-2 cm | *2.513* | *2.655* | *0.040* |
|  | Control | 2-4 cm | 0.691 | -1.171 | 0.645 |
|  | Control | 4-6 cm | 0.577 | -1.319 | 0.551 |
|  | 0-2 cm | 2-4 cm | *0.275* | *-4.128* | *<0.001* |
|  | 0-2 cm | 4-6 cm | *0.230* | *-3.550* | *0.002* |
|  | 2-4 cm | 4-6 cm | 0.836 | -0.462 | 0.967 |
